# Spatial multilevel analysis of individual, household, and community factors associated with COVID-19 vaccine hesitancy in the Dominican Republic

**DOI:** 10.1038/s41598-025-94653-3

**Published:** 2025-04-02

**Authors:** Behzad Kiani, Benn Sartorius, Beatris Mario Martin, Angela Cadavid Restrepo, Helen J. Mayfield, Cecilia Then Paulino, Petr Jarolim, Micheal De St Aubin, Ronald Skews Ramm, Devan Dumas, Salome Garnier, Marie Caroline Etienne, Farah Peña, Gabriela Abdalla, Adam Kucharski, William Duke, Margaret Baldwin, Bernarda Henríquez, Lucia de la Cruz, Eric J. Nilles, Colleen L. Lau

**Affiliations:** 1 University of Queensland Centre for Clinical Research (UQCCR), Faculty of Health, Medicine, and Behavioural Sciences, The University of Queensland, Brisbane, QLD 4029, Australia; 2 School of Public Health, Faculty of Health, Medicine, and Behavioural Sciences, The University of Queensland, Brisbane, Australia; 3 Ministry of Health and Social Assistance, Santo Domingo, Dominican Republic; 4 Brigham and Women’s Hospital, Boston, MA USA; 5 Harvard Medical School, Boston, MA USA; 6 Infectious Diseases and Epidemics Program, Harvard Humanitarian Initiative, Cambridge, MA USA; 7 Department of Infectious Disease Epidemiology and Dynamics, Faculty of Epidemiology and Population Health, London School of Hygiene & Tropical Medicine, London, UK; 8 Faculty of Health Sciences, Pedro Henriquez Urena National University, Santo Domingo, Dominican Republic

**Keywords:** COVID-19, Vaccine hesitancy, Vaccine acceptance, Health belief model, Socioeconomic, Household dynamics, The Dominican Republic, Infectious diseases, Epidemiology

## Abstract

Vaccine hesitancy or refusal poses a significant public health challenge resulting in the resurgence of preventable diseases and undermining the effectiveness of national and global health initiatives. This study investigates multilevel determinants of COVID-19 vaccine hesitancy in the Dominican Republic (DR) shortly after the launch of the national COVID-19 vaccination campaign in February 2021. Participants aged 18 years and older were enrolled through a national multistage cluster survey conducted from June-October 2021. The Health Belief Model guided the selection of potential factors contributing to vaccine hesitancy. Hierarchical mixed-effect logistic regression models were used to examine individual, household, and community factors associated with vaccine hesitancy. COVID-19 vaccine hesitancy was observed in 12.6% (95% CI: 11.7–13.5%) of participants (*n* = 5,566), with spatial variations at the cluster level. Individual factors associated with lower odds of vaccine hesitancy included older age, higher education levels, mulatto ethnicity, and perceiving vaccination as crucial for health. In contrast, factors significantly associated with hesitancy included being born in the DR and concerns about COVID-19 vaccine side effects. For factors at the household level, differential trust in health information sources significantly influenced vaccine hesitancy, with certain sources correlating with increased hesitancy and others with reduced vaccine hesitancy. Better access to healthcare, as indicated by a higher number of hospitals per population, was paradoxically associated with increased vaccine hesitancy. Future strategies to reduce vaccine hesitancy in the DR might consider these multifaceted factors.

**Supplementary Information:**

The online version contains supplementary material available at 10.1038/s41598-025-94653-3.

## Introduction

The COVID-19 pandemic posed unprecedented challenges to global healthcare systems and communities. The rapid development and distribution of safe and effective vaccines became crucial tools in controlling the spread of the virus^1^. However, vaccine hesitancy during the COVID-19 pandemic resulted in reduced vaccine uptake in certain settings. COVID-19 vaccines significantly reduced disease severity, hospitalizations, and deaths, but vaccine hesitancy remains a persistent challenge^2,3^.

Vaccine hesitancy encompasses both delayed and outright refusal of vaccination that manifests differently across locations and social contexts^4^. Individuals’ motivations for vaccination, shaped by beliefs and attitudes towards health, vary widely^5^. Evaluating the risk-benefit perspective requires careful consideration of perceived risks derived from information, such as that circulating through public media and social channels, including concerns about vaccine side effects and exposure to anti-vaccination narratives^6^. Personal experiences such as financial hardship, political affiliations and having chronic medical conditions also contribute to this assessment^7,8^. Importantly, vaccine hesitancy is shaped not only by individual factors but also by household dynamics and the socio-cultural context of communities^9,10^. While an individual’s general opposition to vaccines may increase hesitancy towards COVID-19 vaccines specifically, new vaccines have the potential to introduce unique beliefs about hesitancy that warrant thorough investigation^11^.

Various conceptual frameworks for identifying possible factors associated with vaccine hesitancy elucidate the diverse components influencing health behavior decision-making, including the Health Belief Model (HBM)^12^, theory of planned behavior^13^, Social-Ecological Model^14^, 5As Framework (Access, Affordability, Awareness, Acceptance, and Activation Framework)^15^, COM-B Model (Capability, Opportunity, Motivation, and Behavior Model)^16^, Three Cs Model (Confidence, Complacency, and Convenience Model)^17^, and the 5 C psychological antecedents (confidence, constraints, complacency, calculation, and collective responsibility)^18^. These frameworks highlight the complexity of vaccine hesitancy, encompassing the interplay between psychological, social, cultural, and contextual factors^2,13,19^. For instance, the socio-ecological model was used to examine factors influencing community engagement for general vaccination in India, identifying both enablers and barriers across individual, community, organizational, and policy levels. While supportive policies and social mobilization promoted community engagement, challenges such as limited formal strategies, power imbalances, and insufficient institutional support hindered progress^20^. In Bangladesh, researchers employed the HBM, the Theory of Planned Behaviour, and the 5 C framework of psychological antecedents to examine a range of psychological factors driving COVID-19 vaccine hesitancy. Their findings revealed that the Theory of Planned Behaviour provided the highest predictive accuracy in this context^18^.

The HBM, a psychological framework, has been widely utilized to analyze COVID-19 vaccine hesitancy and and associated determinants^2,18^. The HBM encompasses components such as the perceived severity of and susceptibility to COVID-19, perceived benefits of and barriers to receiving COVID-19 vaccines, and cues to action. These cues can include implicit or explicit incentives or situations that motivate vaccination, such as information from mass media^2^.

Multiple factors shape individuals’ decisions to accept or refuse COVID-19 vaccination, including employment status (e.g., whether a person is employed, unemployed, or retired), religiosity, political affiliation, gender, age, education, ethnicity, and income^2,19,21^. According to recent systematic reviews, primary reasons for vaccine refusal included a general opposition to vaccines, concerns about safety, the perception of COVID-19 as benign, distrust of health authorities, doubts about scientific research and vaccine efficacy, belief in pre-existing immunity, and uncertainty about the vaccine’s origin^8,22^. It is important to note that behavior refers to observable actions or responses, such as deciding whether or not to get vaccinated. In contrast, attitude encompasses an individual’s internal feelings, beliefs, and evaluations regarding a subject. While attitudes can influence behavior, they do not always result in action. For instance, someone who is vaccine hesitant might still need to get vaccinated due to travel restrictions or employment requirements.

While much of the existing research on COVID-19 vaccine hesitancy has focused on high-income countries^8,19^, our study shifts the focus to the Dominican Republic (DR), a middle-income nation with a Gross National Income (GNI) per capita of USD 9,710 in 2023^23^. The DR’s unique sociocultural landscape is shaped by a rich blend of indigenous, African, and European influences, with spirituality and religion playing a central role in shaping societal values and healthcare practices, creating a distinctive approach to health-seeking behaviors and community support systems. Such sociocultural factors, including historical inequalities and varied access to healthcare services, are critical to understanding the context of vaccine hesitancy in the country^24^. Additionally, previous studies have predominantly explored vaccine hesitancy in relation to individual, household, or community-level factors, but rarely all three simultaneously^19^. Previous research conducted in the DR which used a part of this study’s dataset focused narrowly on trust in information sources^25^, while our study addresses this gap by exploring the association of individual, household, and community-level factors with COVID-19 vaccine hesitancy in the DR. Through this approach, we aim to enhance the understanding of COVID-19 vaccine hesitancy in the DR, broaden existing knowledge, and lay the groundwork for identifying contextually relevant factors to reduce vaccine hesitancy for future pandemics.

## Methods

### Theoretical background

We selected the HBM^2^ as the most suitable theoretical framework because it aligned well with our 3-level dataset (namely individual, household and community), and research question. Furthermore, the decision to use the HBM in this study was driven by its robustness in capturing the cognitive factors influencing health behaviours. The HBM’s emphasis on individual perceptions aligns well with the goal of understanding personal decision-making processes regarding vaccination. It allows for a nuanced analysis of how perceived risks and benefits, combined with external cues, influence vaccine acceptance or hesitancy. Table 1 in supplementary materials outlines the categorization of our factors based on the HBM model.

### Study location

**Fig. 1:**
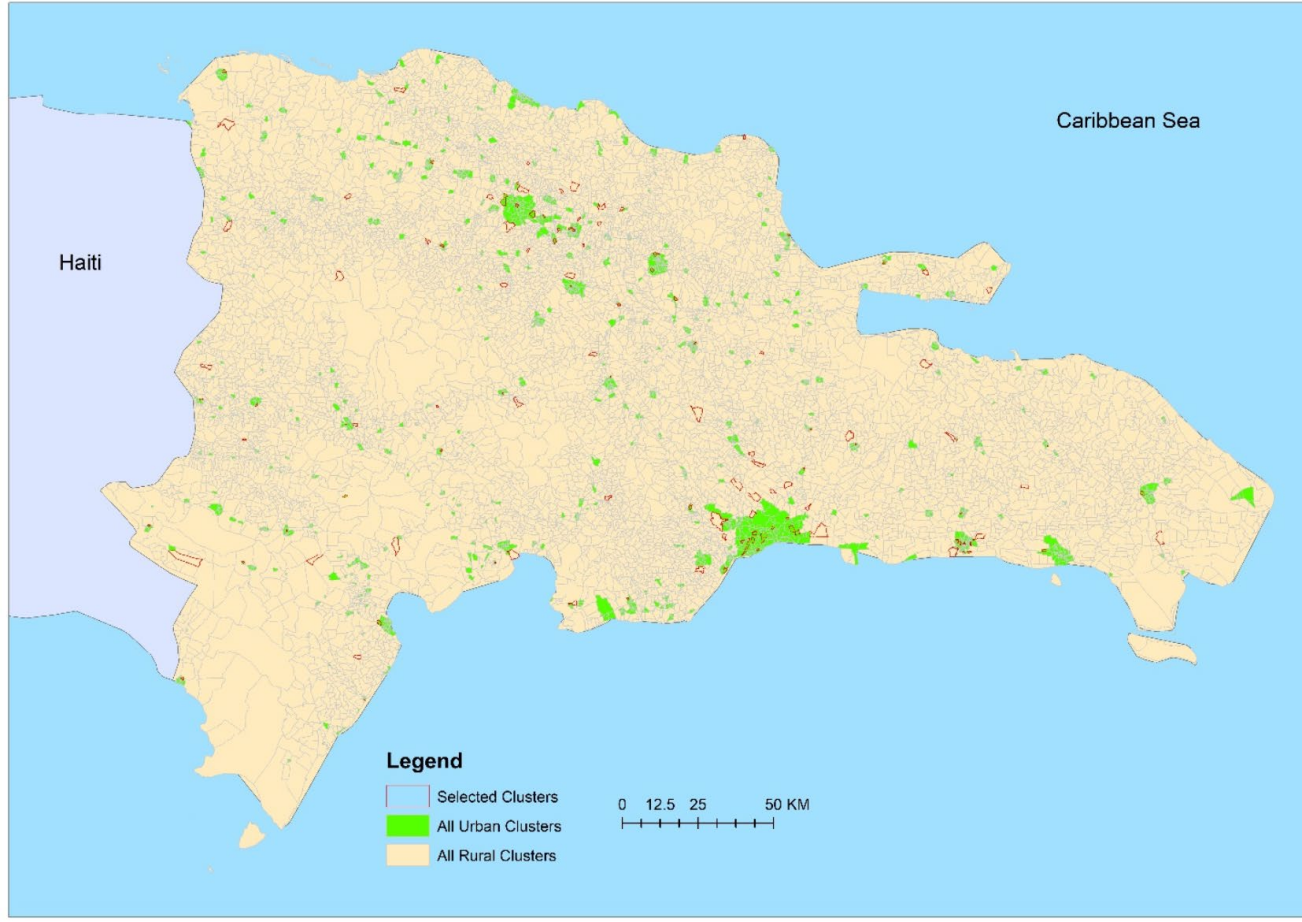
The study area map, including the sampled clusters (outlined by red lines) across the Dominican Republic. The figure was created by the authors using ArcGIS Pro (version 3.4), available at https://www.esri.com/en-us/arcgis/products/arcgis-pro/overview.

The DR, located in the Caribbean on the island of Hispaniola alongside Haiti (Fig. 1), has a population of approximately 10.5 million, making it the second most populous country in the Caribbean. Administrative divisions include 31 provinces and the Santo Domingo National District, comprising 155 municipalities, 386 district municipalities, 1565 sections, and 12,565 barrios/parajes^26^. Barrios refer to neighborhoods or urban communities, while parajes are small rural settlements, similar to unincorporated communities in the United States. Despite 80% of the population residing in urban and semi-urban areas, only about 20% of the total barrios/parajes are designated as urban. Over the past two decades, the DR has witnessed consistent economic growth, leading to an overall reduction in poverty. However, persistent social inequities endure, with higher poverty rates prevalent in urban slums and rural regions, particularly those close to the Haitian border^27^.

### Data sources and sampling

We utilized data collected from a field study in 2021 for the identification of the most common causes of acute febrile illnesses in the DR, which used a spatial random sampling method to select specific clusters (barrios/parajes), across the country (see Fig. 1). In brief, the 31 provinces and the Santo Domingo National District were categorized into five areas for logistical efficiency. Within each area, a predetermined number of urban and rural clusters were chosen utilizing a spatially representative sampling method. In urban clusters, a grid approach was employed for household selection^28^. Conversely, in rural clusters, households were selected using a spatially representative sampling method designed to optimize spatial dispersion, thereby preventing oversampling in both densely populated and sparsely populated regions^29^.

 Sampled households and individuals were nested within primary sampled clusters across the country, and we collected detailed information at both the individual and household levels^30^ using a two-part questionnaire. The first section, administered to all participants, included individual-level questions covering areas such as demographics, personal health status and vaccination attitudes. Household-level questions addressed shared circumstances, such as household environment (electricity, water, toilets, etc.) and whether any household member had died from COVID-19. Household representatives were selected by the household, generally one of the adults capable of responding to questions on their behalf. The structure of the questionnaire was therefore well suited to an analysis at the individual and household levels.

 A total of 23 households per cluster were chosen in 132 clusters, with an exception for two provinces, which were associated with a study on clinical surveillance of acute febrile illnesses^31^. In these two provinces, each cluster was oversampled with 60 households. All household members aged ≥ 5 years were invited to participate in the national survey. However, for the purposes of the current study, we only included individuals aged 18 years or older, i.e. adults. Individuals less than 18 years of age were excluded as they would typically not play a decisive role in vaccine uptake decision-making.

Additionally, we used open-source datasets, such as motorized travel time to the nearest health facility and poverty index, both available in a raster format^32^ and census data presented in Table 1 in the supplementary file, to obtain information at the community level. To extract the values, we matched the geographic coordinates of households with the corresponding pixels in the raster files.

### Outcomes

The primary outcome of interest was COVID-19 vaccine hesitancy. To capture nuanced attitudes towards COVID-19 vaccination, we adopted a two-pronged approach in our analysis, including both binary and ordinal variables as outcomes. By employing both models, we aimed to gain a comprehensive understanding of vaccine hesitancy, accounting for both a simplified binary categorization and the spectrum of hesitancy levels.

We created a binary vaccine hesitancy variable by categorizing participants’ responses to the question, “If it were available to you right now, would you accept a COVID-19 vaccine?” with “No, definitely not,” “Probably not,” “Don’t know/Not sure,” and “Probably yes,” as indicating hesitancy, and “Yes, definitely” or “Already vaccinated” as not hesitant. We included “Probably yes” in the hesitancy category because every degree of uncertainty or reluctance reflects a form of vaccine hesitancy, which we believe should be captured in the binary model. This approach ensures that even the mildest forms of hesitancy are accounted for, as they still represent potential barriers to vaccination.

Additionally, we assessed vaccine hesitancy as an ordinal variable to acknowledge varying degrees of hesitancy. Responses ranged from strong hesitancy (“No, definitely not”, “Probably not” and " Don’t know/Not sure”) to mild hesitation (“Probably yes”), and no hesitancy (“Yes, definitely” or “Already vaccinated”). This approach allowed us to capture the spectrum of participants’ attitudes towards the vaccine.

While this study focuses on findings related to the binary outcome classification, results for the ordinal outcome have been provided in Supplementary Table 2.

## Data analysis

### Cluster analysis

Spatial Scan Statistics, using SaTScan version 10.1.3, were employed to identify clusters with high or low levels of vaccine hesitancy^33^. The Bernoulli model, implemented in SaTScan, allows for the detection of clusters by comparing the observed distribution of binary outcomes (vaccine hesitancy, in this case) to what would be expected under the assumption of spatial randomness.

### Variables selection for modelling

To determine the appropriateness of each predictor for inclusion in our 3-level hierarchical mixed effect models, we calculated bivariate associations between each pair of potential predictors and both outcomes. Variables with a p-value < 0.05 were included in our models. To ensure the robustness of our multilevel modeling approach, we conducted a comprehensive assessment of potential associations among the predictor variables as follows.

Variance Inflation Factors (VIFs) were computed to investigate potential correlations among continuous/ordinal variables, and a value above 7 was considered indicative of strong correlation. These variables were categorized based on their conceptual meaning. If high VIF values were observed within each group, a more general variable was selected based on the researchers’ judgment. For instance, the variables “the degree of concern regarding the impact of COVID-19 on an individual’s health” and “the degree of concern regarding the impact of COVID-19 on family health” exhibited elevated VIFs, each having a value equal to or greater than 7, which is considered notably high. Consequently, only the former variable was included in the modeling process.

For categorical variables, we calculated Cramér’s V, which measures chi-squared effect size, to determine the strength of association between different categorical predictors. We did not observe any strong associations, as all values were less than 0.3.

### Three-level modelling

To quantify the role of each variable in vaccine hesitancy in the DR, we employed a three-level mixed-effect multivariable logistic regression modelling approach due to the hierarchical structure of our data. Specifically, individuals were nested within households, and households within clusters. We utilized a generalized linear modelling approach since our outcomes were ordinal and binary. StataSE (version 18, College Station, etc.) was used for statistical modeling, and R 4.3.2 for data preparation.

The regressions incorporated weights derived from the survey design, considering the sampling process in three stages. Initially, the likelihood of a cluster being selected was computed by taking into account the total number of clusters within each region, and the corresponding weight was defined as the inverse of the selection probability for that category. Subsequently, the probability of a household being selected was determined based on the total number of households in each cluster, and the associated weight was the inverse of the household selection probability. In the final stage, the weights from the first two steps were multiplied and adjusted for a finite population. These sampling weights at each level were incorporated into our models. A comprehensive explanation of the weight calculation is available in a recently published work^26^.

We initially included all the predictors selected based on VIF and bivariate associations with the outcome in the hierarchical model. The final model included only the variables that were significant in the initial model at p-value < 0.05.

## Results

### Descriptive statistics

Table 1 shows the descriptive statistics for independent variables included in our models. The mean age of the participants was 46 years (standard deviation 17.85). Most participants were born in the DR (95.49%). Ethnicity varied, with mulatto (51.68%) and mestizo (33.44%) groups being the predominant categories. Most participants reported having completed primary or secondary school (36.67% and 36.20% respectively). Participants primarily reported a mixed work environment including both indoor and outdoor (15.32%).

**Table 1:** Descriptive statistics of characteristics of adults living in the Dominican Republic (surveyed June-October 2021).

Category	Variable (level)	Descriptive statistics		
Sociodemographic factors	Age (individual)	Mean	Standard deviation	
		46.68	17.85	
	The degree of poverty in the community household located (community)^*^	47.37	14.67	
	Born in the Dominican Republic (individual)	Value	Number	Percentage
		Yes	5,318	95.49
		No	230	4.13
		Don’t know or refuse	18	0.32
		NA	3	0.05
	Ethnicity (individual)	Indigenous	681	12.23
		Mestizo	1,862	33.44
		Mulatto^*^	2,878	51.68
		White	124	2.23
		Don’t know or refuse	10	0.18
		NA	3	0.05
		Other	11	0.20
	Education (individual)	No formal	681	12.23
		Primary	2,042	36.67
		Secondary	2,016	36.20
		Technical	110	1.98
		University	699	12.55
		Don’t know or refuse	18	0.32
		NA	3	0.05
	Work environment (individual)	Indoor	654	11.74
		Mix	853	15.32
		Not relevant (e.g. student or not working)	3,549	63.73
		Outdoor	513	9.21
	Travel in the past 5 years outside of the country (individual)	Yes	278	4.99
		No	5,284	94.88
		Refuse to answer	4	0.07
		NA	3	0.05
	Home toilet (household)	Inside	4,277	76.80
		Outside	1,228	22.05
		No toilet	55	0.99
		Don’t know	9	0.16
	Setting (household)	Urban	2,988	53.65
		Rural	2,581	46.35
Health related factors	Death outside of the household (individual)	Value	Number	Percentage
		Yes	905	16.25
		No	4,597	82.55
		Don’t know or refuse	64	1.15
		NA	3	0.05
	Death inside the household (household)	Yes	256	4.60
		No	5,149	92.46
		Refuse to answer	7	0.13
		Don’t know	157	2.82
	Number of past/current chronic diseases (individual)	0	3,739	67.14
		1	1,442	25.89
		2	361	6.48
		3	24	0.43
		4	3	0.05
	Number of past/current infectious diseases (individual)	0	3,469	63.32
		1	1,920	34.50
		2	161	2.89
		3	16	0.29
	How concerned are you about the impact on your health if you contract coronavirus? (individual)	Very low	1,578	28.35
		Low	1,169	21
		Average	935	16.80
		High	1,264	22.71
		Very high	620	11.14
Vaccine-related factors	Vaccinating myself against COVID-19 is important for my health (individual)	Value	Number	Percentage
		Strongly disagree	64	1.15
		Almost disagree	157	2.82
		Neutral	432	7.76
		Agree	1,048	18.83
		Strongly agree	3,865	69.44
	The benefits of vaccination against COVID-19 outweighs the risks (individual)	Strongly disagree	77	1.38
		Almost disagree	288	5.17
		Neutral	580	10.42
		Agree	1,084	19.48
		Strongly agree	3,537	63.55
	The benefits of general vaccination (childhood vaccination, or other adult vaccination) outweigh the risks (household)	Strongly disagree	39	0.70
		Almost disagree	124	2.23
		Neutral	442	7.94
		Agree	1,071	19.23
		Strongly agree	3,893	69.90
	Newer vaccines are as safe as older vaccines (household)	Strongly disagree	34	0.62
		Almost disagree	202	3.63
		Neutral	462	8.30
		Agree	1,113	19.99
		Strongly agree	3,758	67.48
	I am concerned about serious adverse effects of the COVID-19 vaccine (individual)	Very low	3,152	56.63
		Low	798	14.34
		Average	933	16.76
		High	190	3.41
		Very high	493	8.86
	I am concerned about serious adverse effects of vaccines (individual)	Very low	2,971	53.35
		Low	525	9.43
		Average	933	16.75
		High	506	9.09
		Very high	634	11.38
Main source of health information, this includes but is not limited to information about COVID-19. (household)		Value	Number	Percentage
		None	38	0.68
		Social media	1,742	31.30
		School	130	2.34
		Internet	343	6.16
		Health professionals	460	8.27
		Media	2,732	49.09
		Friends and relatives	120	2.16
Trust in general	The local doctors/local clinic (household)	Value	Number	Percentage
		Very low	23	0.41
		Low	107	1.92
		Average	347	6.23
		High	1,122	20.15
		Very high	3,970	71.29
	The local government (household)	Very low	94	1.69
		Low	211	3.79
		Average	593	10.65
		High	1,540	27.65
		Very high	3,131	56.22
	The religious leaders (household)	Very low	61	1.10
		Low	266	4.78
		Average	586	10.52
		High	1,395	25.05
		Very high	3,261	58.56
	The media (household)	Very low	67	1.20
		Low	166	2.98
		Average	667	11.98
		High	1,496	26.86
		Very high	3,173	56.98
	The social media (household)	Very low	208	3.73
		Low	357	6.41
		Average	771	13.84
		High	1,462	26.25
		Very high	2,771	49.76
	The national government (household)	Very low	168	3.02
		Low	306	5.49
		Average	698	12.53
		High	1,388	24.92
		Very high	3,009	54.03
	Scientists (household)	Very low	235	4.22
		Low	333	5.98
		Average	598	10.74
		High	1,253	22.50
		Very high	3,150	56.56
	Global health institutes like the World Health Organization (household)	Very low	484	8.69
		Low	335	6.02
		Average	601	10.80
		High	1,024	18.39
		Very high	3,125	56.11
Healthcare access factor	Number of hospitals per 100,000 population in the community household located (community)	Mean	Standard Deviation	
		227.3	206.43	

Mulatto: the first-generation offspring of a Black person and a white person.

The degree of poverty in the community household located: it is global poverty relative index.

(Floating point index from 0 to 100, where a value of 100 represents the highest level of relative deprivation and a value of 0 the lowest.). It has been referenced in the supplementary file.

Regarding health-related factors, the majority (56.63%) expressed little concern about the serious adverse effects of the COVID-19 vaccine. Attitudes towards vaccination revealed nuanced perspectives: a significant proportion strongly agreed that vaccinating against COVID-19 is important for their health (69.44%) and that its benefits outweigh the risks (63.55%). Trust in various sources of health information exhibited similar patterns, with a notable skepticism towards information obtained from social media.

### Cluster analysis

**Fig. 2:**
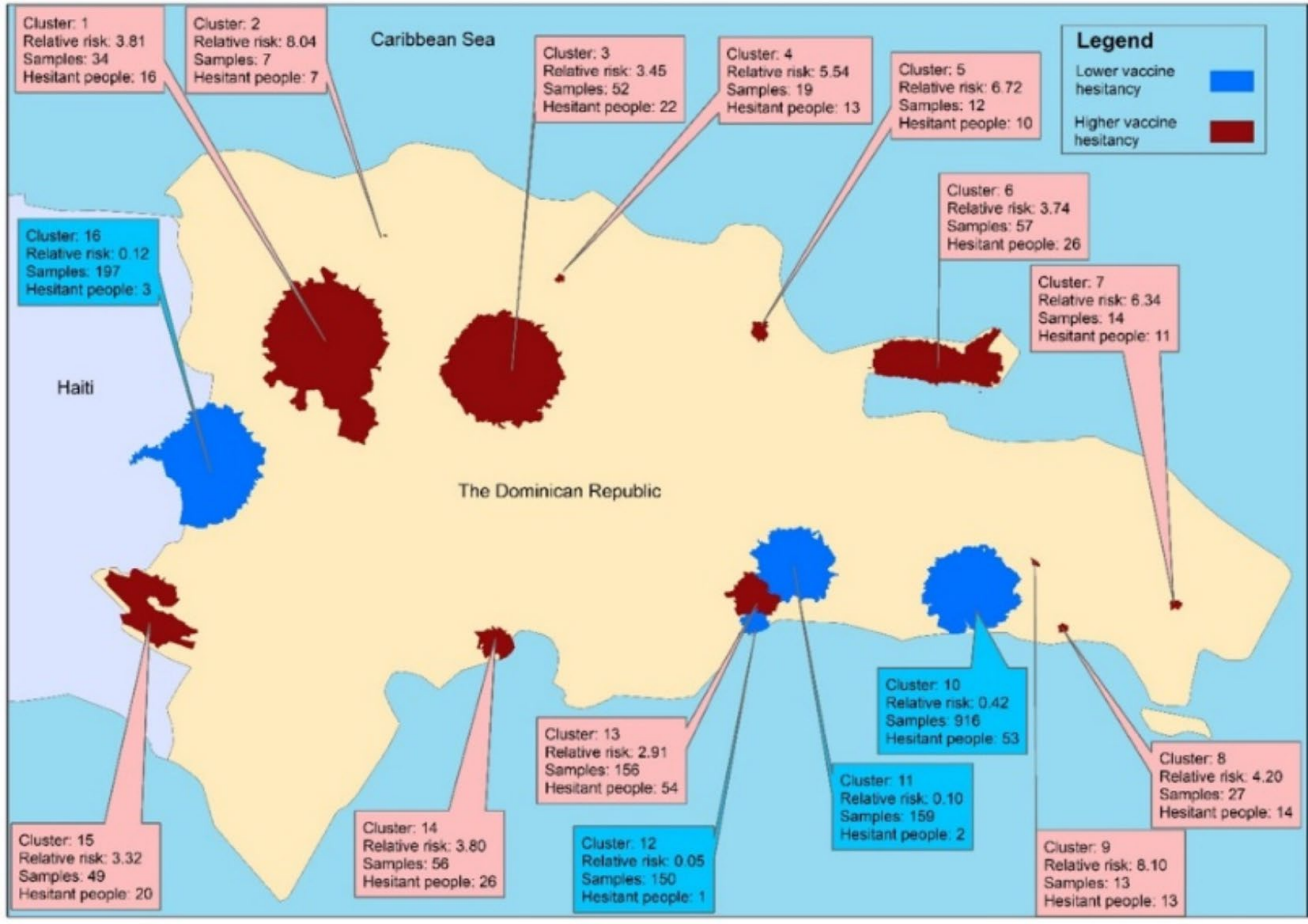
Geographic clustering of COVID-19 vaccine hesitancy and readiness among adults in the Dominican Republic (surveyed June-October 2021). All clusters are statistically significant at *P* < 0.05. The figure was created by the authors using ArcGIS Pro (version 3.4), available at https://www.esri.com/en-us/arcgis/products/arcgis-pro/overview.

Cluster analysis using SaTScan revealed 16 statistically significant clusters of lower (*n* = 4) and higher (*n* = 12) vaccine hesitancy across the DR. Notably, the study identifies clusters 2 (North-central, near the coast) and 9 (Southeast, near the coast) as areas where all individuals were hesitant to COVID-19 vaccination (Fig. 2).

### Modeling results

 Among 5566 adults surveyed in DR, vaccine hesitancy was 12.6% (*n* = 699 participants) (95% CI: 11.7–13.5%).

 The multilevel model identified several significant factors associated with COVID-19 vaccine hesitancy. Older age was associated with decreased odds of being vaccine hesitant, with odds ratio (OR) = 0.47 (95% CI: 0.28–0.80) for each year of age increase. Mulatto ethnicity was associated with a significantly reduced odds of being vaccine hesitant when compared to Caucasians, (OR = 0.11, 95% CI: 0.02–0.66). Notably, those born outside the DR exhibited higher levels of vaccine hesitancy, (OR = 43.67, 95% CI: 6.01-317.22). Education played a crucial role, with higher educational attainment associated with reduced hesitancy, particularly among those who completed secondary school, (OR = 0.17, 95% CI: 0.05–0.61) or university, (OR = 0.18, 95% CI: 0.03–0.95) compared to those did not have any formal education.

 Health-related factors, such as the perceived importance of vaccinating against COVID-19 for personal health, were found to be inversely associated with COVID-19 vaccine hesitancy (OR = 0.21, 95% CI: 0.09–0.49). However, higher concern regarding the adverse effects of the COVID-19 vaccine was positively associated with COVID-19 vaccine hesitancy (OR = 4.34 (95% CI: 2.15 8.78).

 The sources of health information were also associated with COVID-19 vaccine hesitancy. For instance, individuals who relied on guidance from health professionals and schools demonstrated lower levels of vaccine hesitancy compared to those who reported that they obtained information from social media.

Trust in local government appeared to be associated with less COVID-19 vaccine hesitancy (OR = 0.68, 95% CI: 0.47–0.98), while trust in religious leaders (OR = 2.86, 95%CI: 1.04–7.85) and the media (OR = 1.93, 95% CI: 1.28–2.90) associated with higher COVID-19 vaccine hesitancy.

Regarding community characteristics, the number of hospitals per population was associated with higher COVID-19 vaccine hesitancy (OR = 1.10, 95% CI: 1.06–1.14).

Finally, the random effects analysis revealed variance of COVID-19 vaccine hesitancy at both the household and community levels, emphasizing the importance of considering these contextual factors using hierarchical modelling. Table 2 in the supplementary file also shows the results of modelling using ordinal variables as the outcome.

**Table 2:** Factors associated with COVID-19 vaccine hesitancy based on the 3-level hierarchical model in the Dominican Republic (surveyed June-October 2021).

Predictors	Odds ratio (95% CI)	*P* value
Intercept	0.20 (0.03–1.36)	0.100
Age	0.47 (0.28–0.80)**	0.005
Ethnicity (reference: white)		
Indigenous	0.49 (0.13–1.82)	0.289
Mestizo	0.24 (0.03–1.69)	0.151
Mulatto	0.11 (0.02–0.66)**	0.016
Born in the Dominican Republic (reference: yes) no	43.67 (6.01-317.22)**	< 0.001
Education level (reference: no formal)		
Primary school	0.40 (0.14–1.13)	0.084
Secondary School	0.17 (0.05–0.61)**	0.006
Technical	0.62 (0.06–6.37)	0.688
University	0.18 (0.03–0.95)**	0.043
Work environment: (reference: indoor)		
Mix	1.31 (0.46–3.74)	0.608
Not relevant	1.08 (0.48–2.43)	0.854
Outdoor	0.41 (0.10–1.70)	0.220
Travel in the past 5 years outside of the country (reference: yes) no	0.30 (0.07–1.19)*	0.087
Vaccinating myself against COVID-19 is important for my health	0.21 (0.09–0.49)**	< 0.001
I am concerned about serious adverse effects of the COVID-19 vaccine	4.34 (2.15 8.78)**	< 0.001
The benefits of vaccination against COVID-19 outweighs the risks	0.62 (0.36–1.08)*	0.089
Death in the household from any cause (reference: no) yes	0.54 (0.18–1.58)	0.258
Source of health information (reference: social media)		
Health professionals	0.30 (0.10–0.92)**	0.035
Internet	1.88 (0.18–20.10)	0.600
Neighbors, family/friends, co-workers	4.13 (0.77-22.00)*	0.097
school	0.08 (0.01–0.99)**	0.050
TV, radio, newspapers, brochures	1.77 (0.66–4.74)	0.253
Trust the religious leaders	2.86 (1.04–7.85)**	0.042
Trust the media	1.93 (1.28–2.90)**	0.002
Trust the local government	0.68 (0.47–0.98)**	0.039
Trust the social media	0.94 (0.63–1.38)	0.738
Trust the national government	1.22 (0.87–1.70)	0.254
Number of hospitals per population in the community household located	1.10 (1.06–1.14)**	0.000
Variance and covariance of random effects		
Level 2 (Household)	6.41 (2.74)	
Level 3 (Community)	2.31 (0.67)	
Log-Likelihood	-1737636.2	

“*” indicates a significant association at *P* < 0.1, and “** “indicates a significant association at *P* < 0.05.

## Discussion

This study suggests a low level of vaccine hesitancy in the DR and identified multifaceted factors associated with COVID-19 vaccine hesitancy among adults in the DR. Our analysis reveals a complex interplay of demographic, socio-economic, health-related, and informational determinants linked to COVID-19 vaccine hesitancy. These factors underscore the critical need for targeted public health strategies to reduce vaccine hesitancy that takes into account factors such as age, ethnicity, educational attainment, perceived health benefits, concerns about adverse effects, sources of health information, and trust in authorities. Interestingly, our analysis revealed that better access to healthcare, indicated by a higher number of hospitals per population, was paradoxically associated with increased COVID-19 vaccine hesitancy. This counterintuitive finding suggests that in areas with better healthcare access, other factors, such as trust in the healthcare system or exposure to misinformation, might play a more significant role in influencing vaccine decisions. Additionally, spatial clustering, as well as variability observed within household and community contexts highlights the necessity of localized and context-sensitive approaches in tackling COVID-19 vaccine hesitancy and specific regions where targeted intervention efforts may be particularly warranted.

Spatial analysis revealed significant heterogeneity as well as clustering of vaccine hesitancy (or lower levels thereof) across different regions in the DR. Higher vaccine hesitancy was observed predominantly in the north-central, east-central, and southeast coastal areas, with some very small clusters where all sampled individuals were hesitant. Conversely, lower vaccine hesitancy was concentrated in the northwest and south-central regions. This geographical variation underscores the need for location-specific strategies to reduce vaccine hesitancy. Factors contributing to these patterns may include social selection, where individuals with similar sociodemographic traits cluster together, and social influence, where vaccine attitudes spread within communities^34^. Targeted interventions in high-hesitancy areas could involve localized education campaigns and community engagement efforts to foster vaccine acceptance.

Our analysis revealed several key findings on COVID-19 vaccine hesitancy in the DR. Sociodemographic factors, particularly older age and mulatto ethnicity, were associated with lower COVID-19 vaccine hesitancy. Conversely, younger people, appeared to exhibit higher hesitancy, potentially influenced by negative social media narratives or a belief in their own resilience^35^. Individuals born outside the DR also exhibited higher COVID-19 vaccine hesitancy, suggesting that this group might not fully trust the country’s health system^36^. Mistrust may stem from negative past experiences with healthcare, language barriers, and cultural differences, leading to poor communication with providers^37,38^. Limited access to reliable vaccine information and opportunities, potentially due to systemic inequities, further exacerbates the issue^39^. Solutions could include community outreach, culturally sensitive education, and policies to reduce access barriers for immigrants.

Educational attainment played a crucial role, with lower hesitancy observed among those with secondary or university education compared to those with no formal education. Previous research has shown higher education to be a significant predictor of COVID-19 vaccine acceptance across multiple countries, highlighting the need for tailored public health messaging and interventions that consider diverse educational backgrounds^40^. Future research should investigate contextual factors to deepen our understanding of the influences on vaccine hesitancy in the Dominican Republic and other countries in the region.

Consistent with the HBM, health-related beliefs appear to be significantly associated with COVID-19 vaccine hesitancy. Participants who perceived vaccination as crucial for personal health were less likely to be hesitant, whereas those with concerns about serious adverse effects of the COVID-19 vaccine exhibited higher hesitancy. This highlights the importance of addressing misconceptions and fears about vaccine safety through effective public health communication. Previous research has shown that vaccinated individuals often expressed strongly positive views regarding the safety and effectiveness of COVID-19 vaccines and considered community benefits, whereas unvaccinated individuals often expressed neutral or negative views on vaccine safety and effectiveness​^40^.

The role of information sources and trust also appeared to be contextually important correlates of vaccine hesitancy. Reliance on health information from social media was associated with higher COVID-19 vaccine hesitancy, which aligns with previous research^41^. Our analysis revealed that trust in local government and health professionals was associated with lower vaccine hesitancy, while higher trust in religious leaders and the media correlated with increased hesitancy. These findings are consistent with the only previous study conducted in the DR^25^, which also utilized a portion of this dataset. However, even with the introduction of additional variables in our study—spanning individual, household, and community levels—trust remained a significant predictor of vaccine hesitancy. This reinforces the critical role of trust in shaping public health outcomes, particularly in the context of the DR. The robustness of these trust-related factors across different analytical models highlights their importance, suggesting that effective public health campaigns must prioritize building and maintaining trust in reliable sources while actively countering misinformation from less trustworthy channels. A randomized controlled trial conducted in the United States found that even a brief exposure to an infographic explaining a scientific process can enhance trust in science, potentially influencing individuals’ willingness to adopt COVID-19 preventive measures^42^. Furthermore, engaging religious health leaders to address their concerns about vaccines could help reduce vaccine hesitancy, both among the leaders themselves and the people who trust them.

A significant strength of this study is the application of the HBM model as the theoretical framework, which guided the selection of variables most relevant to understanding vaccine hesitancy. By focusing on perceptions of vaccine side effects and beliefs about the severity and susceptibility to COVID-19—factors often overlooked in prior research in the DR—we provide a more nuanced understanding of vaccine hesitancy. Additionally, our inclusion of community-level factors, such as motorized travel time to the nearest health facility and the number of hospitals per community household, adds an important layer of analysis that has been largely absent in earlier studies. This multi-level approach, combined with the broader range of individual and household-level variables, offers a more comprehensive view of the factors influencing vaccine hesitancy.

## Limitations

This study has several limitations. Eligible individuals who refused to participate may have had higher levels of COVID-19 vaccine hesitancy than study participants. Moreover, the data were collected through self-reporting, which may have introduced social desirability bias. While based in existing theory, the scale used to measure COVID-19 vaccine hesitancy was not a validated tool, as it was developed in response to the rapidly changing circumstances of the pandemic and evolving vaccine availability. We considered individuals who had already received the COVID-19 vaccine as non-hesitant. This operational definition may overlook cases where individuals harbor vaccine hesitancy yet got vaccinated due to external pressures, such as employment or travel requirements. Although the Dominican Republic did not implement a universal mandate for COVID-19 vaccination, specific circumstances might have influenced some to vaccinate despite personal reservations. As a result, our findings should be interpreted with caution, acknowledging that vaccination behaviour may not always perfectly reflect underlying attitudes.

Another limitation involves the use of different geographical scales for data extraction at the community level; for example, illiteracy rates were obtained at the municipality level, while poverty levels were derived from a raster dataset intersected with household locations, which may introduce variability in our community-level findings. Furthermore, in the extracted raster data of Poverty, Development Threat Index, and Motorized Travel Time to the nearest healthcare facility, some null values were identified in the exact household locations. To estimate these null values, we utilized the average value of the four nearest pixels with non-null values.

Additionally, the definition and reporting of ethnicity in the DR can vary due to differences in how individuals self-identify and how ethnicity is categorized in different datasets. While this variability could introduce some degree of uncertainty, we believe that including ethnicity in our model still provides valuable insights. The potential inconsistencies are unlikely to significantly affect the overall findings, but future research could benefit from more standardized and reliable measures of ethnicity to further enhance the accuracy of such analyses.

## Conclusion

This study highlights the complex and multifaceted landscape of COVID-19 vaccine hesitancy among adults in the DR, identifying significant associations with demographic, socio-economic, health-related, and informational factors. The findings underscore the critical need for targeted public health strategies such as enhancing vaccine education, fostering trust in healthcare providers, and addressing socio-economic disparities. The observed spatial clustering and community level factors emphasizes the importance of localized interventions tailored to regional contexts. Our study suggests that future efforts should focus on these areas to effectively address and reduce COVID-19 vaccine hesitancy in DR and serve as a model for other countries in the region or with similar local context. This study can also inform future public health interventions for pandemic response, beyond COVID-19.

## Electronic supplementary material

Below is the link to the electronic supplementary material.

Supplementary Material 1

## Data Availability

The data presented in this study are available on request from the corresponding author. The data are not publicly available due to preserving participants’ privacy.
