# Supplementary material for: Spatial multilevel analysis of individual, household, and community factors associated with COVID-19 vaccine hesitancy in the Dominican Republic
Source: Sci Rep. 2025 Apr 2;15:11203. doi: 10.1038/s41598-025-94653-3 (PMC11961636; doi:10.1038/s41598-025-94653-3)
Supplement: Supplementary file 1 — Supplementary Material 1 [file 41598_2025_94653_MOESM1_ESM.docx]

Supplementary materials:

Table 1: Data elements used according to Health Belief Model to study associations of individual, household, and community level factors with COVID-19 vaccine hesitancy in the Dominican Republic during the pandemic (June-October 2021)

| Health Belief Model component | DR Survey Question / Open Dataset Indicator | | | Coding for modelling purpose | Included in models? | Data level | Source |
| --- | --- | --- | --- | --- | --- | --- | --- |
| Perceived susceptibility | 1-Chronic disease history:  (High blood pressure, coronary heart disease/prior heart attack, diabetes, cancer, kidney disease, stroke, asthma, chronic obstructive airways disease, disease of the immune system (does not include seasonal allergies) | | | Continuous  (Number of chronic diseases) | Yes | individual | DR survey |
|  | 2-Infectious disease history:  (Dengue, Zika, Chikungunya, Leptospirosis Typhoid) | | | Continuous  (Number of infectious diseases) | Yes | individual | DR survey |
| Perceived severity | 3-How concerned are you about the impact on your health if you contract coronavirus?  (Not at all concerned=1, slightly concerned=2, somewhat concerned=3, moderately concerned=4, extremely concerned=5) | | | Continuous  (As indicated) | Yes | individual | DR survey |
|  | 4-How concerned are you about the impact on your family if you contract coronavirus?  (Not at all concerned=1, slightly concerned=2, somewhat concerned=3, moderately concerned=4, extremely concerned=5) | | | Continuous  (As indicated) | No (highly correlated with number #3) | individual | DR survey |
| Perceived benefits | General vaccination | | 5- Vaccinating my child is important for my child’s health  (1= strongly disagree, 2= somewhat disagree, 3= neutral, 4= somewhat agree, 5= strongly agree) | Continuous  (As indicated) | No (highly correlated with number #6) | household | DR survey |
|  |  |  | 6- The benefits of vaccination outweigh the risks  (1= strongly disagree, 2= somewhat disagree, 3= neutral, 4= somewhat agree, 5= strongly agree) | Continuous  (As indicated) | Yes | household | DR survey |
|  | COVID vaccination | | 7- Newer vaccines are as safe as older vaccines  (1= strongly disagree, 2= somewhat disagree, 3= neutral, 4= somewhat agree, 5= strongly agree) | Continuous  (As indicated) | Yes | household | DR survey |
|  |  |  | 8- Vaccinating myself against COVID is important for my health  (1= strongly disagree, 2= somewhat disagree, 3= neutral, 4= somewhat agree, 5= strongly agree) | Continuous  (As indicated) | Yes | individual | DR survey |
|  |  |  | 9- Vaccinating myself against COVID is important for my family's health  (1= strongly disagree, 2= somewhat disagree, 3= neutral, 4= somewhat agree, 5= strongly agree) | Continuous  (As indicated) | No (highly correlated with number #8) | individual | DR survey |
|  |  |  | 10- Vaccinating myself against COVID is important for the health of others in my community  (1= strongly disagree, 2= somewhat disagree, 3= neutral, 4= somewhat agree, 5= strongly agree) | Continuous  (As indicated) | No (highly correlated with number #8) | individual | DR survey |
|  |  |  | 11- The benefits of vaccination against COVID outweighs the risks  (1= strongly disagree, 2= somewhat disagree, 3= neutral, 4= somewhat agree, 5= strongly agree) | Continuous  (As indicated) | Yes | individual | DR survey |
| Perceived barriers | General vaccination | | 12- I am concerned about serious adverse effects of vaccines:  (1= strongly disagree, 2= somewhat disagree, 3= neutral, 4= somewhat agree, 5= strongly agree) | Continuous  (As indicated) | Yes | household | DR survey |
|  | COVID vaccination | | 13- I am concerned about serious adverse effects of the COVID vaccine:  (1= strongly disagree, 2= somewhat disagree, 3= Neutral, 4= somewhat agree, 5= strongly agree) | Continuous  (As indicated) | Yes | individual | DR survey |
|  | 14- Motorized travel time to nearest health facility (minutes) | | | Continuous  (As indicated) | No (bivariate association with the outcome was not significant) | community | Open data  (1) |
|  | 15- Does someone in the household own motorized vehicle: (yes, no)  Reference: yes | | | Categorical  (As indicated) | No (bivariate association with the outcome was not significant) | household | DR survey |
| Cues to actions | 16- Do you personally know anyone outside your household who died of COVID?  (Yes, no, don’t know/prefer not to say)  Reference: no | | | Categorical  (As indicated) | Yes | individual | DR survey |
|  | 17- How likely is it that you previously had COVID?  (Very unlikely=1, unlikely=2, don't know=3, likely=4, very likely=5, Certain=6) | | | Continuous  (As indicated) | No (bivariate association with the outcome was not significant) | individual | DR survey |
|  | 18- Have you been in close contact with anyone with LABORATORY CONFIRMED coronavirus?  (Yes, no, don’t know/prefer not to say)  Reference: no | | | Categorical  (As indicated) | No (bivariate association with the outcome was not significant) | individual | DR survey |
|  | 19- Did anyone in your household die of COVID?  (Yes, no, don’t know, prefer not to say)  Reference: no | | | Categorical  (As indicated) | Yes | household | DR survey |
|  | 20- Media as a source of knowledge against COVID-19  (none=0, social media such as WhatsApp, Facebook, Twitter, Instagram, Snapchat, etc.=1, school=2, internet (non-social media) =3, health professionals=4, TV, radio, newspapers, and brochures=5, neighbors, family/friends, co-workers, relatives in U.S=6, Government officials=7, other=8 )  Reference: none | | | Categorical  (As indicated) | Yes | household | DR survey |
|  | 21- Trust the local doctors/local clinic  (Not at all=1, not very much=2, neutral=3, little=4, a lot=5) | | | Continuous  (As indicated) | Yes | household | DR survey |
|  | 22- Trust the local government  (Not at all=1, not very much=2, neutral=3, little=4, a lot=5) | | | Continuous  (As indicated) | Yes | household | DR survey |
|  | 23- Trust the religious leaders  (Not at all=1, not very much=2, neutral=3, little=4, a lot=5) | | | Continuous  (As indicated) | Yes | household | DR survey |
|  | 24- Trust the media (radio, TV, newspapers)  (Not at all=1, not very much=2, neutral=3, little=4, a lot=5) | | | Continuous  (As indicated) | Yes | household | DR survey |
|  | 25- Trust the social media (WhatsApp, Facebook, Twitter, Instagram, Snapchat)  (Not at all=1, not very much=2, neutral=3, little=4, a lot=5) | | | Continuous  (As indicated) | Yes | household | DR survey |
|  | 26- Trust the national government  (Not at all=1, not very much=2, neutral=3, little=4, a lot=5) | | | Continuous  (As indicated) | Yes | household | DR survey |
|  | 27- Trust the scientists  (Not at all=1, not very much=2, neutral=3, little=4, a lot=5) | | | Continuous  (As indicated) | Yes | household | DR survey |
|  | 28- Trust the global health institutions (e.g., WHO, PAHO)  (Not at all=1, not very much=2, neutral=3, little=4, a lot=5) | | | Continuous  (As indicated) | Yes | household | DR survey |
| Modifiers | 29- Gender (male, female, other, prefer not to say)  Reference: male | | | Categorical  (As indicated) | No (bivariate association with the outcome was not significant) | individual | DR survey |
|  | 30- Place of birth (DR, Other)  Reference: DR | | | Categorical  (As indicated) | Yes | individual | DR survey |
|  | 31- Person’s ethnicity  (Caribbean-European (Mulatto), African-Carribean (Mestizo), indigenous, white, other, don’t know/prefer not to say)  Reference: white | | | Categorical  (As indicated) | Yes | individual | DR survey |
|  | 32- Education  (No formal schooling, primary (1-8), secondary (9-12), technical/vocational school, university/tertiary, don’t know/prefer not to say)  Reference: no formal schooling | | | Categorical  (As indicated) | Yes | individual | DR survey |
|  | 33- Smoking (Current, non-smoker)  Reference: non-smoker | | | Categorical  (As indicated) | No (bivariate association with the outcome was not significant) | individual | DR survey |
|  | 34- Body mass index (BMI)  (BMI < 18.5) = “underweight”, (BMI >= 18.5 and BMI <25) = “normal weight", (BMI >= 25 and BMI <30) = “overweight”, (BMI>=30) ="Obese"  Reference: “normal weight” | | | Categorical  (As indicated) | No (bivariate association with the outcome was not significant) | individual | DR survey |
|  | 35- Past 5 years travel history outside of the DR  (Yes, no, prefer not to say)  Reference: no | | | Categorical  (As indicated) | Yes | individual | DR survey |
|  | 36- Occupation  (Unemployed, house person, active worker, retired, student)  Reference: unemployed | | | Categorical  (As indicated) | No (bivariate association with the outcome was not significant) | individual | DR survey |
|  | 37- Work environment  (Indoor work, outdoor work, mix)  Reference: Indoor work | | | Categorical  (As indicated) | Yes | individual | DR survey |
|  | 38-Personal hygiene | 38-1) The last time that you used the bathroom at home, did you clean your hands afterwards? (Yes, no, don’t know/prefer not to say) | | (Number of “yes” answered” in these three questions)  Continuous  (As indicated) | No (bivariate association with the outcome was not significant) | individual | DR survey |
|  |  | 38-2) The last time that you ate a meal at home, did you clean your hands before eating? (Yes, no, don’t know/prefer not to say) | |  |  |  |  |
|  |  | 38-3) The last time that you blew your nose, coughed, or sneezed into your hands, did you clean your hands afterwards? (Yes, no, don’t know/prefer not to say) | |  |  |  |  |
|  | 39- Does this house have piped water (city water supply)? (Yes, no)  Reference: yes | | | Categorical  (As indicated) | No (bivariate association with the outcome was not significant) | household | DR survey |
|  | 40- Toilet type in the house:  (Toilet inside the house, toilet outside the house (e.g., pit latrine, composting toilet), no toilet (e.g., use bucket or bush))  Reference: toilet inside the house | | | Categorical  (As indicated) | Yes | household | DR survey |
|  | 41- Household illiteracy rate  (Number of adults without formal school per total number of adults) | | | Continuous (As indicated) | No (bivariate association with the outcome was not significant) | household | DR survey |
|  | 42- Household unemployment rate  (Number of unemployed adults per total number of adults) | | | Continuous (As indicated) | No (bivariate association with the outcome was not significant) | household | DR survey |
|  | 43- Municipality adult illiteracy rate | | | Continuous (As indicated) | No (bivariate association with the outcome was not significant) | community | census data |
|  | 44- Municipality unemployment rate  (Number of unemployment adults per total number of adults) | | | Continuous (As indicated) | No (bivariate association with the outcome was not significant) | community | census data |
|  | 45- Global poverty relative index  (Floating point index from 0 to 100, where a value of 100 represents the highest level of relative deprivation and a value of 0 the lowest.) | | | Continuous  (As indicated) | Yes | community | Open data  (2) |
|  | 46- Setting  (Rural/Urban)  Reference: Urban | | | Categorical  (As indicated) | Yes | household | DR survey |
|  | 47- Population density  (Number of people per km^2^) | | | Continuous  (As indicated) | No (bivariate association with the outcome was not significant) | community | Open data  (3) |
|  | 48- The ratio of the number of public primary healthcare facilities to the population | | | Continuous  (As indicated) | No (bivariate association with the outcome was not significant) | community | Open data  (4) |
|  | 49- The ratio of the number of education facilities to the population | | | Continuous  (As indicated) | No (bivariate association with the outcome was not significant) | community | Open data  (5) |
|  | 50- Access to Pipe Water  (The ratio of the number of houses having access to pipe water per total of houses in the municipality) | | | Continuous  (As indicated) | No (bivariate association with the outcome was not significant) | community | DR survey |
|  | 51- No access to inside toilet  (The ratio of the number of houses without toilet inside per total number of houses in the municipality) | | | Continuous  (As indicated) | No (bivariate association with the outcome was not significant) | community | DR survey |
|  | 52- The ratio of the number of hospitals to the population | | | Continuous  (As indicated) | Yes | community | Open data  (6) |

"*" indicates a significant association at P < 0.1, and "** "indicates a significant association at P < 0.05.

Table 2: Final 3-Level Hierarchical Models for Predictors of COVID-19 Vaccine Hesitancy as an ordinal variable in the Dominican Republic during the pandemic (June-October 2021)

| Predictors | Odds ratio (95% CI) | |
| --- | --- | --- |
| Intercept | cut11 | 3.50 (-0.97-7.96) |
|  | cut12 | 6.37 (0.43-12.31) |
| Age | 0.47 (0.26 - 0.88)** | |
| Ethnicity (Reference: white) |  | |
| indigenous | 0.52 (0.09 - 3.08) | |
| mestizo | 0.43 (0.05 - 3.83) | |
| mulatto | 0.25 (.03 - 1.94) | |
| Born in Dominican Republic: (Reference: Yes)  No | 106.10 (1.78 - 6306.82)** | |
| Education level (Reference: no formal) |  | |
| Primary School | 0.28 (0.05 - 1.73) | |
| Secondary School | 0.13 (0.02 - .73)** | |
| Technical | 0.37 (0.07 - 1.90) | |
| University | 0.20 (0.02 - 2.20) | |
| Work environment: (Reference: Indoor) |  | |
| Mix | 1.07 (0.26 - 4.54) | |
| Outdoor | 0.34 (0.04 - 2.79) | |
| Travel in the past 5 years outside of the country: (Reference: Yes)  No | 0.32 (0.05 - 2.25) | |
| Vaccinating myself against COVID is important for my health | .19 (.06 - .58)** | |
| I am concerned about serious adverse effects of the COVID vaccine | 8.67 (1.31 - 57.41)** | |
| The benefits of vaccination against COVID outweighs the risks | 0.45 (0.16 - 1.27) | |
| Source of health information: (Reference: social media) | | |
| Health professionals | 0.35 (0.06 - 1.90) | |
| Internet | 4.75 (0.44 - 51.74) | |
| Neighbors, Family/Friends, Co-workers | 19.80 (0.27 - 1467.59) | |
| School | 0.29 (0.02 - 5.34) | |
| TV, Radio, Newspapers, Brochures | 1.93 (0.68 - 5.52) | |
| Trust the religious leaders | 3.38 (0.81 - 14.07)* | |
| Trust the media | 2.74 (0.91 - 8.28)* | |
| Trust the local government | 0.43 (0.16 - 1.20) | |
| Trust the social media | 0.83 (0.46 - 1.49) | |
| Number of hospitals per population in the community household located | 1.23 (1.01 - 1.50) | |
| Variance and covariance of random effects |  | |
| Level 2 (Household) | 13.84 (12.92) | |
| Level 3 (Community) | 4.01 (3.65) | |
| Log-Likelihood | -2502406.5 | |

"*" indicates a significant association at P < 0.1, and "** "indicates a significant association at P < 0.05.

**References:**

1. Weiss DJ, Nelson A, Vargas-Ruiz CA, Gligorić K, Bavadekar S, Gabrilovich E, et al. Global maps of travel time to healthcare facilities. Nature Medicine. 2020;26(12):1835-8.

2. University CfIESIN-C-C. Global Gridded Relative Deprivation Index (GRDI), Version 1. Palisades, New York: NASA Socioeconomic 2022 [cited 2024 29 July]. Available from: <https://doi.org/10.7927/3xxe-ap97>.

3. Center for International Earth Science Information Network CCU. Gridded Population of the World, Version 4 (GPWv4): Population Density Adjusted to Match 2015 Revision UN WPP Country Totals, Revision 11. Palisades, New York: NASA Socioeconomic Data and Applications Center (SEDAC); 2018.

4. Team HO. Dominican Republic Health Facilities (OpenStreetMap Export) 2024 [Available from: <https://data.humdata.org/dataset/hotosm_dom_health_facilities>.

5. Team HO. Dominican Republic Education Facilities (OpenStreetMap Export) 2024 [Available from: <https://data.humdata.org/dataset/hotosm_dom_education_facilities>.

6. HDX. Dominican Republic Healthsites 2024 [Available from: <https://data.humdata.org/dataset/dominican-republic-healthsites>.
